# Donald F. Steiner, MD, 1930–2014: Pioneering diabetes researcher

**DOI:** 10.1007/s00125-015-3495-x

**Published:** 2015-01-24

**Authors:** Kenneth S. Polonsky, Arthur H. Rubenstein

**Affiliations:** 1 Department of Medicine, University of Chicago, 5841 S Maryland Ave, Chicago, IL 60637 USA; 2 Department of Medicine, Raymond and Ruth Perelman School of Medicine, University of Pennysylvania, Philadelphia, PA USA


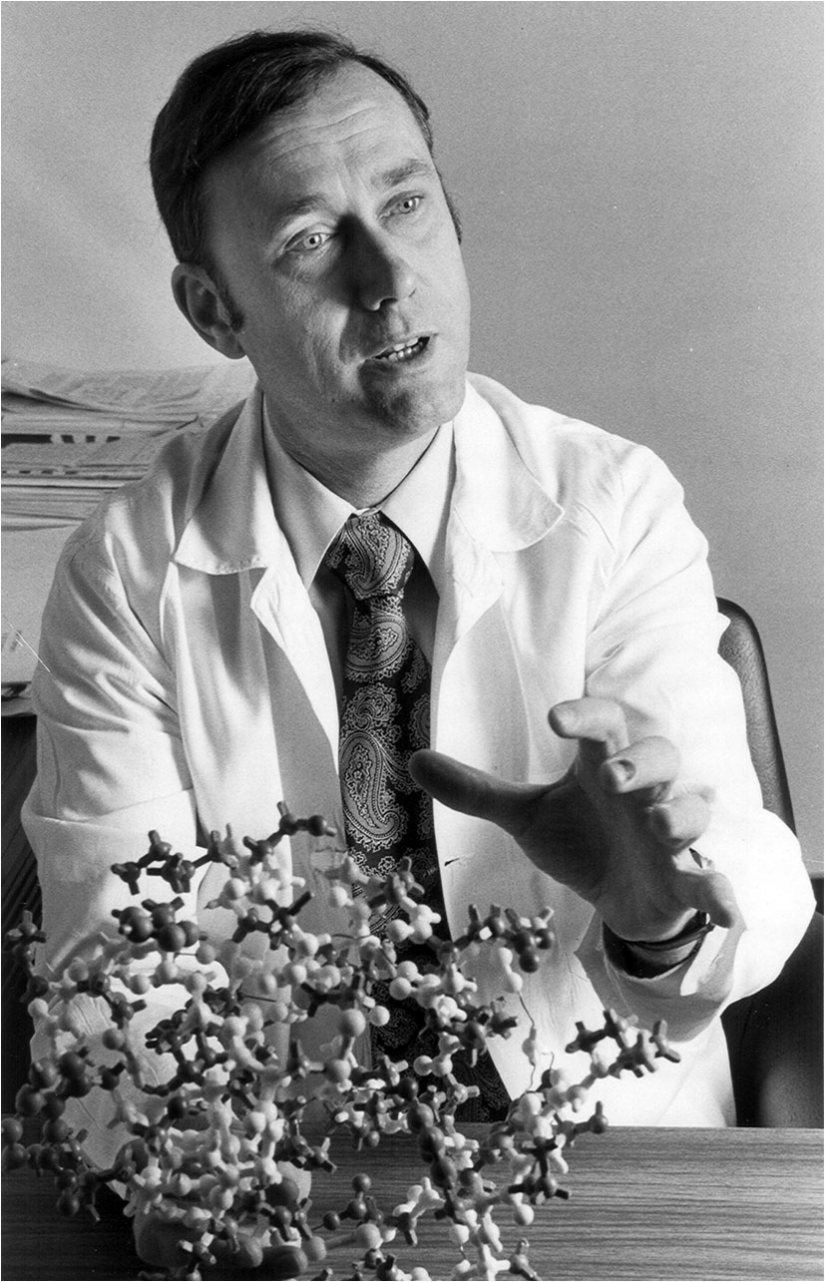


Donald Frederick Steiner, MD, a scientist whose research fundamentally changed our view of insulin production, died from natural causes at his home in Chicago on Tuesday, 11 November 2014. He was 84 years old, and had been a member of the University of Chicago faculty since 1960.

Steiner, the A.N. Pritzker Distinguished Service Professor Emeritus in the Departments of Medicine and Biochemistry & Molecular Biology at the University of Chicago, revolutionised thinking about insulin synthesis and secretion. In 1967, he showed that insulin, thought to be made from two separate protein chains, was derived from a single chain precursor, which he named ‘proinsulin’. Steiner showed how a small interior part of that single long chain was removed by proteolytic processing leaving behind two chains (A and B) connected by disulphide bonds, which together comprise the insulin molecule. The interior segment that was removed was termed ‘C-peptide’ for ‘connecting peptide’.

Like so many breakthroughs, the discovery of proinsulin began with a prepared mind and a stroke of luck. In September 1965, Steiner learned that a gastrointestinal surgeon at the University of Chicago was about to perform a resection on a patient with an insulinoma. Steiner called to request a sample and the surgeon agreed.

‘When I got the tumor tissue,’ Steiner later recalled, ‘I decided to see what I could find out.’ He froze the tissue for 6 weeks while he read up on various methods of working with pancreatic tissue [1].

He started his experiments on 24 October 1965 and soon found that, in addition to insulin, the cells also produced a larger protein related chemically and immunologically to insulin. When he exposed the larger protein to the enzyme trypsin, it was converted to a protein that was indistinguishable from insulin. These findings were published in a paper in *Proceedings of the National Academy of Sciences* in February 1967 [2]. Subsequently Steiner discovered a larger precursor that he called ‘preproinsulin’. In 1968, a team of scientists at Eli Lilly, although initially sceptical, confirmed the finding. Within 10 years, Steiner and others found that this mechanism of proteolytic processing of a large single chain precursor to form the smaller mature peptide applied to many other hormones and neuropeptides. Steiner’s discovery was therefore broadly applicable throughout biology.

This fundamental discovery paved the way to understanding how other hormones, such as glucagon, as well as neuropeptides in the brain and endocrine system, are made and processed, thus establishing the field of protein-precursor processing. It enabled the pharmaceutical industry to improve the purity of insulin preparations, leading to insulins that were less likely to provoke an immune response, and paved the way for biosynthetic human insulin production.

This was, by anyone’s standards, a remarkable piece of work—a truly creative and ultimately beautiful set of experiments. No one else at that time, his colleagues insist, was thinking through such problems in the same way. Steiner’s careful and imaginative work has impacted on millions of people who have been treated with insulin.

Steiner gave generously of his time and expertise to colleagues, collaborators and trainees. It was well known that when junior colleagues went to seek advice about a scientific problem they would have the benefit of his complete attention and the breadth of his knowledge and experience. Steiner loved to talk about science and to think about how to solve scientific problems in creative ways.

Dr Rudolph Leibel, a director of the Naomi Berrie Diabetes Center at Columbia University College of Physicians and Surgeons, told the *New York Times* that the discovery of proinsulin ‘opened a window into a whole area of molecular physiology that people were not aware of.’ Despite his fundamental discoveries, he added, Dr Steiner ‘was very modest, and very concerned about young people—his students and his co-workers. He’s from that era, these great thinkers who were soft-spoken and gentlemanly and not in it for anything but the joy of doing the science. We don’t have as much of that now’ [3].

The discovery of proinsulin has also had substantial implications for understanding the physiology of insulin secretion and for clinical medicine. Thus measurement of C-peptide in the bloodstream provides a very accurate indication of how much insulin is produced by the body, and this has been extensively used as an independent indicator of insulin secretion in clinical studies and in clinical trials. Circulating proinsulin has also been used for the diagnosis of insulin-secreting tumours of the pancreas and has also been shown to be an early marker of pancreatic beta cell dysfunction in the evolution of diabetes.

In 1983, 16 years after his ground-breaking paper on proinsulin, Steiner was part of a University of Chicago team that described the first mutations in the insulin gene, now known as ‘insulin Chicago’ [4]. Such mutations—we now know more than 30—are associated with syndromes that combine mild diabetes and elevated circulating insulin or proinsulin. Other mutations in the insulin gene have been associated with neonatal diabetes. Steiner also contributed to work on understanding how insulin binds to its receptor and on insulin receptor mutations.

Donald F. Steiner was born in Lima, Ohio, on 15 July 1930. Colleagues said his small-town roots shaped his personality. He grew up in a family with a strong belief in honesty, generosity and hard work, with respect for everyone. That, together with his scientific brilliance, made him the perfect mentor. He taught the people he trained how to do research and then gave them the credit for doing what he taught them. That’s one reason so many good people flocked to his lab, where he only made them better.

Steiner earned his BS in chemistry and zoology from the University of Cincinnati in 1952, followed by an MS in biochemistry and an MD from the University of Chicago in 1956. He completed his internship at King County Hospital in Seattle, followed by a residency and post-doctoral fellowship at the University of Washington. He was invited to return to the University of Chicago as an assistant professor of biochemistry in 1960.

He rose quickly through the ranks, becoming a full professor in 1968 and department chairman in 1973. From 1985 to 2006 he was a senior investigator in the Howard Hughes Medical Institute at the University of Chicago. He also served as director of the University of Chicago Diabetes and Endocrinology Center (1974–1978), as well as associate director (1977–1981), director (2000–2004) and co-director (2004–2014) of the University of Chicago Diabetes Research and Training Center. In those roles, he realised: ‘I could get along with anyone… but basically, I am not interested in administration’ [1].

He preferred to use his talents planning and performing research, and teaching, and that paid off. Steiner published more than 450 peer-reviewed papers. His work has been cited more than 10,000 times by other researchers. He won dozens of prestigious national and international honours and awards, often several per year. These include the Lilly Award and the Banting Medal for Scientific Achievement from the American Diabetes Association, the Joslin Medal from the New England Diabetes Association, Israel’s Wolf Prize [5] and, from Japan, the Manpei Suzuki International Prize for Diabetes Research [6]—the largest financial award for diabetes research. When granting him the Borden Award, the Association of American Medical Colleges said his ‘discovery of proinsulin is widely regarded as one of the most exciting basic science discoveries in the past two decades’ [7].

He was elected to membership of the American Academy of Arts & Sciences in 1972, the National Academy of Sciences in 1973, honorary membership of the European Association for the Study of Diabetes in 2001, and the American Philosophical Society, the USA’s oldest learned society, in 2004.

In addition to his seminal contributions to science, Don Steiner has had a profound influence at the University of Chicago, particularly on the diabetes programme. The broad implications of his discoveries of the pathways of insulin biosynthesis and secretion placed the University of Chicago at the forefront of diabetes research. In 2014, in appreciation of all he had done for the institution, Dr Steiner was awarded the University of Chicago Alumni Medal [8].

Steiner also was an avid patron of the arts, especially the Chicago Symphony Orchestra. He helped support large and small theatre companies and the opera. He was a talented pianist. He played classical music on his Steinway grand piano—an acquisition made possible by the Wolf Prize—in his high-rise apartment overlooking Lake Michigan. In the late 1960s, while picking apart proinsulin, he meticulously assembled a harpsichord in his basement laboratory at the University. He also enjoyed sailing on Lake Michigan and gardening.

Because of Donald Steiner, we all know a lot more about diabetes, and people with diabetes live better lives. The authors of this remembrance came to the University of Chicago to work with him. He was an extraordinary mentor for us and for many people, combining professional brilliance with the drive to solve hard problems and the capacity, once the job was done, to share the credit. He was a model of how science should be done and how a friend should behave.

The atmosphere in his laboratory was pleasant, but the planning, execution and evaluation of experiments was rigorous and meticulous. He spent hours worrying about unexpected results and repeated experiments numerous times before being satisfied. We all felt part of his team and when new discoveries were made, we all shared in the joy of the science and celebrated together. Remarkably, he never spoke negatively of others, even when they were critical or sceptical of his work; rather, he was generous in his praise. This was admired and respected by colleagues all over the world.

Despite his awards and accomplishments, Steiner was an extraordinarily kind, humble, gentle and attentive person. He always had time for his staff and colleagues, would answer questions at length and in depth, and was absolutely devoted to this University. We shall miss him profoundly.

Don Steiner is survived by Ellen Steiner, the wife of his late brother Phares, by his niece Adrienne Steiner, his nephew Paul Steiner, several cousins and many, many friends.

A memorial service is scheduled for 1 May 2015 at the University of Chicago.
